# Intra-Articular Hyaluronic Acid Compared to Traditional Conservative Treatment in Dogs with Osteoarthritis Associated with Hip Dysplasia

**DOI:** 10.1155/2016/2076921

**Published:** 2016-10-26

**Authors:** Gabriel O. L. Carapeba, Poliana Cavaleti, Gabriel M. Nicácio, Rejane B. Brinholi, Rogério Giuffrida, Renata N. Cassu

**Affiliations:** ^1^Postgraduate Program in Animal Science, Oeste Paulista University, 19067-175 Presidente Prudente, SP, Brazil; ^2^Faculty of Veterinary Medicine, Oeste Paulista University, 19067-175 Presidente Prudente, SP, Brazil

## Abstract

The purpose of this study was to compare the efficacy of the intra-articular (IA) hyaluronic acid injection to traditional conservative treatment (TCT) in dogs with osteoarthritis (OA) induced by hip dysplasia. Sixteen dogs were distributed into two groups: Hyal: IA injection of hyaluronic acid (5–10 mg), and Control: IA injection with saline solution (0.5–1.0 mL) in combination with a TCT using an oral nutraceutical (750–1000 mg every 12 h for 90 days) and carprofen (2.2 mg/kg every 12 h for 15 days). All dogs were assessed by a veterinarian on five occasions and the owner completed an assessment form (HCPI and CPBI) at the same time. The data were analyzed using unpaired *t* test, ANOVA, and Tukey's test (*P* < 0.05). Compared with baseline, lower scores were observed in both groups over the 90 days in the veterinarian evaluation, HCPI, and CPBI (*P* < 0.001). The Hyal group exhibited lower scores from 15 to 90 and 60 to 90 days, in the CBPI and in the veterinarian evaluation, respectively, compared to the Control group. Both treatments reduced the clinical signs associated with hip OA. However, more significant results were achieved with intra-articular hyaluronic acid injection.

## 1. Introduction

Hip dysplasia (HD) represents one of the orthopedic diseases with the highest incidence in the canine species, characterized by abnormal development of the hip joint, the etiology of which is multifactorial [1, 2]. HD is a degenerative joint disease that can progressively trigger the development of osteoarthritis (OA) of the affected joint [3], characterized by articular cartilage lesions, bone remodeling with the presence of osteophytes and inflammation [4].

The most common symptom of OA is joint pain, which involves peripheral and central sensitization mechanisms. The peripheral sensitization is triggered by injury and/or joint inflammation, which results in the release of neurotransmitters such as bradykinin, prostaglandins E2 and I2, serotonin, and leukotrienes. These primary neuromediators stimulate the release of neuropeptides such as calcitonin gene-related peptide (CGRP) and substance P in the lesion site [5]. The persistent and prolonged inflammatory stimulus results in central sensitization, which is related to hyperexcitability of spinal cord neurons and other central nervous system structures [6]. In addition to inflammation, the accumulation of stressors on the joint (e.g., overweight, joint instability, and excessive exercise) favors the destruction of cartilage, triggering alterations in the articular surface remodeling, synovial membrane changes, and an increased synovial fluid with decreased viscosity and lubrication properties [7, 8].

Although there is no curative intervention at present, the pharmacological treatment for OA is principally palliative and aims to relieve pain and improve function of the affected joint. In dogs, one of the principal conservative therapeutic approaches involves oral administration of nutraceuticals, whose formulation is primarily composed of glucosamine and chondroitin sulfate together with the use of nonsteroids anti-inflammatory drugs (NSAIDs) [9, 10]. However, prolonged use of NSAIDs can be associated with side effects, especially in the digestive system and kidneys [11], so complementary and alternative medicine is increasingly offering concomitant therapeutic options.

Hyaluronic acid (HA) is a naturally occurring glycosaminoglycan and a component of synovial fluid and cartilage matrix. The molecular properties of HA favor viscosity and lubrication of cartilage, essential factors for proper joint performance [12]. In cases of degenerative joint disease drastic reduction in the concentration of HA occurs, compromising the viscosity of the synovial fluid [13]. Thus, one of the causes of pain and mobility impairment of the joint appears to be associated with the decreased protective effect of this viscoelastic medium on the pain receptors in the synovial tissue [14].

The preparations of HA currently available may be classified according to their molecular weight (MW) and formulation type, solutions of low MW (500–730 kDa), solutions of intermediate MW (800–2000 kDa), solutions of high MW (6000 kDa), cross-linked HA, and solutions of nonanimal stabilized HA (NASHA) [13]. The majority of exogenous HA remains in the joint for a few days; however, the clinical therapeutic effects of HA treatment may be seen between 5 and 13 weeks after injection, although improvements have also been observed after 14–26 weeks and sometimes even longer [15].

In humans, intra-articular (IA) injection of HA is an increasingly popular therapy for knee OA. Several clinical trials have reported the beneficial effects following IA injection of HA, with reduction in pain and improvement in joint function [15–17]. However, few studies have evaluated the clinical efficacy of HA administered through IA in the management of hip OA in man [18, 19].

The purpose of this study was to compare the clinical efficacy of intra-articular use of hyaluronic acid to TCT (nutraceutical/NSAID) administered orally in dogs with OA of the hip joint. The hypothesis of the study is to examine whether intra-articular HA injection is superior to TCT in pain relieve. The time expected for the maximal effect was at least 30 days after HA injection.

## 2. Methods

The study was performed following the guidelines of the Brazilian College of Animal Experimentation, and the experimental procedure was approved by the Institutional Animal Care Committee (protocol 1611-CEEA).

### 2.1. Animals

From August 2014 to July 2015, 16 client-owned dogs with HD/OA were enrolled. All procedures were performed with written consent of the owners. The age of the screened dogs varied from 1.5 to 15 years (12 ± 5 years) and the body weight from 07 to 35 kg (20.7 ± 7.8 kg). The study only included dogs with chronic pain (signs of pain for a period of at least three months) which had not been given any type of analgesic drug (NSAIDs or corticosteroids) or nutraceutical for at least eight weeks. In addition, the dogs only participated in the study if their owners reported at least two common clinical signs of dogs with HD/OA, such as difficulty in lying down or getting up, difficulty in jumping or refusing to jump, difficulty in going up or down stairs, or lameness. Animals were excluded if they presented lameness of both thoracic and pelvic limbs, presented lameness of neurologic etiology, showed neurological deficits, were pregnant, had a history of recurrent gastritis/gastric ulcers, or had severe systemic diseases. If OA of another major joint was suspected clinically, additional radiographs were also made.

The dogs included in the study (*n* = 16) were evaluated by laboratory tests (complete blood count, serum urea, creatinine, alanine aminotransferase enzymes, aspartate aminotransferase, and alkaline phosphatase) prior to initiation of treatment and monthly during the evaluation period (90 days).

### 2.2. Physical and Radiographic Evaluation

At the first evaluation, clinical data including age, weight, sex, duration of symptoms, previous or concomitant diseases, and current medications were recorded. As body weight may influence the clinical response to treatment, this variable was measured monthly. The severity of clinical signs was measured by a single veterinarian (blinded to treatment allocation) using an ordinal scoring system (Table 1) which included pain on palpation, the ability to jump and climb stairs, lameness, and stiffness of movements [20, 21].

A radiographic examination was performed to confirm the HD/OA. For correct positioning for the radiographic examination, the dogs were sedated with a combination of 0.03 mg/kg of acepromazine maleate 0.2% (Acepran, Vetnil, Brazil) and 0.5 mg/kg of morphine (Dimorf, Cristália, Brazil) intramuscularly (IM). In cases of insufficient analgesia for the correct positioning of the animal, intravenous (IV) propofol (Propovan, Cristália, Brazil) was administered to effect. Radiographic evaluations included the ventrodorsal and lateral views following the standards set by the* Orthopedic Foundation for Animals* (OFA). The degree of HD was measured according to the standard established by evaluation of hip radiographs, in which coxofemoral joints were classified into 5 classes, from A to E [22, 23]. Category A represents normal joints, while category E represents the most severely affected joints. All the dogs included in this study were classified as grade D or E. The radiological criteria of joint OA severities used in this study were based on the Takahashi scoring system [citar]: grade 0 (normal) = not affected; grade I (mild) = doubtful narrowing of joint space and possible osteophytic lipping; grade II (moderate): definite osteophytes and possible narrowing of joint space; grade III (severe): moderate multiple osteophytes, definite narrowing of joints space, some sclerosis, and possible deformity of bone contour; and grade IV (very severe): large osteophytes, marked narrowing of joint space, severe sclerosis, and definite deformity of bone contour [24].

### 2.3. Treatments

In a double-blind, controlled clinical trial the dogs were randomly assigned to receive IA hyaluronic acid (Hyal, *n* = 8) or saline solution (Control, *n* = 8). A random number generator (Research Randomizer, computer software, https://www.randomizer.org/) was used to assign the dogs to each of the two groups with block randomization. For the treatment allocation, the dogs received consecutive numbers based on the order of enrollment in the study. After the dogs were entered into the study by a veterinary surgeon, the assigned numbers were sent to a research assistant who prepared the injections according to the numbers received.

Each dog in the Hyal group received an IA injection of hyaluronic acid (Hyalovet, Hetacarpe, Brazil) at doses of 5 mg (animals weighing less than or equal to 10 kg) or 10 mg (animals weighing more than or equal to 11 kg) in both affected joints. The formulation injected was composed of purified HA, with a low MW (500–730 kDa) and a concentration of 10 mg/mL; it was not cross-linked by a chemical agent. An equivalent volume of 0.9 per cent saline solution was administered to the Control group. In addition, the dogs in the Control group were treated with an oral nutraceutical (Condroton, Vetnil, Brazil; every 12 h for 90 days) and carprofen (Rymadil, Bayer, Brazil; 2.2 mg/kg every 12 h for 15 days). For the nutraceutical, the dosing regimen was 750 and 1000 mg, respectively, for dogs weighting 10.0–20.0 and 21.0–50.0 kg. The nutraceutical formulation contained glucosamine, chondroitin sulfate, and collagen.

The dogs were positioned in lateral recumbency with the affected hip uppermost. The hair over the lateral aspect of the hip was clipped and the skin aseptically prepared. The articular access was guided by ultrasound, with the animals under inhalational anaesthesia. Arthrocentesis was performed through a craniolateral approach and confirmed through aspiration of synovial fluid. The hip infiltration was performed with the patient anaesthetized. All dogs were premedicated with IM acepromazine maleate 0.2% (0.03 mg/kg) associated with morphine (0.5 mg/kg), both in the same syringe. Twenty min later, the cephalic vein was catheterized and general anaesthesia was introduced using propofol. Orotracheal intubation was performed, and anaesthesia was maintained with isoflurane (Isoforine, Cristália, Brazil) in oxygen using a small animal rebreathing circuit (SAT 500, Takaoka, Brazil). Lactated Ringer's solution was administered at 10 mL/kg/h until recovery.

Twenty-four hours after the IA injection, the owners were asked by telephone to provide information on the behaviour of their dog during this period including questions on the presence of skin irritation or discomfort (such as licking or biting the injection site, any increase in the degree of lameness, greater difficulty in getting up or lying down, or the occurrence of vocalization).

### 2.4. Owner Assessment

In addition to the periodic evaluations performed by the veterinarian, the dogs were evaluated by the owners at home using two descriptive questionnaires: Helsinki Chronic Pain Index (HCPI) [25] and the Canine Brief Pain Inventory (CBPI) [26, 27] including the total pain scores (TPS) and those referring to pain severity scores (PSS) and pain interference scores (PIS).

Clinical improvement was associated with a decrease of at least 30% in the overall CBPI and/or HCPI posttreatment scores in comparison with the pretreatment values.

The evaluations performed by the veterinarian and the owners of the dogs were carried out prior to treatment (baseline) and 15, 30, 60, and 90 days after the IA injection. During the evaluation period (90 days), systemic analgesic therapy (carprofen, 2.2 mg/kg every 12 h for one week) was permitted in cases where the sum of the scores evaluated by the owner exceeded 50% of the total possible value evaluated by the CBPI and/or HCPI. In the case of alterations in the digestive system, such as appetite loss, anorexia, and/or episodes of vomiting, concomitant oral administration of the gastric mucosa protector, omeprazole (0.17 mg/kg every 24 hours), was permitted.

### 2.5. Adverse Effects

The manifestation of adverse effects such as pain, the presence of bruising at the injection site, the occurrence of vomiting, or diarrhea was evaluated.

### 2.6. Outcome Measures

The primary outcome measures were the CBPI and HCPI pain scales. Secondary outcome measures included veterinary assessment score, quality of life, and requirement for the rescue analgesia.

### 2.7. Statistical Analysis

The sample size was estimated to be a minimum of eight animals per group for a power test of 80%, alpha level of 5%, and a standard deviation (SD) of 12 to identify a reduction in the CBPI scores of 30% compared to baseline. SD was estimated from a pilot study.

The data were submitted to the Shapiro-Wilk and Kolmogorov-Smirnov normality tests to identify the distribution. For the variables weight, age, duration of symptoms, and overall clinical improvement, the unpaired *t* test was used to compare the groups. The sum of the scores evaluated by the veterinarian and the owners (CBPI and HCPI) was evaluated through analysis of variance (ANOVA) and Tukey's test to compare differences between groups and differences over time within the same group. Relationships between age, weight, and primary outcome measures at baseline and after treatment were examined using the Pearson Rank correlation statistic. The differences between treatments at each time, differences in time for each treatment, and interaction between treatment were performed using analysis of variance with the *F* test followed by Tukey's test using IBM SPSS Statistics Graphpad software. A *P* value less than 0.05 was considered significant (Table 2).

## 3. Results

A total of 40 dogs were screened to obtain 18 dogs eligible for inclusion in this study. Of the 22 dogs excluded, 18 dogs did not meet the inclusion criteria because of back pain associated with neurologic deficit (*n* = 04) and no hip OA, lack of OA in hip joints on radiographs (*n* = 07), lack of pain on manipulation of hip joints (*n* = 3), and abnormal laboratory results (*n* = 4). In addition, four of the owners refused to participate in the study. Two dogs were excluded from the study after enrollment because their owners did not complete the evaluations posttreatment.

There were no significant differences between groups in demographic and baseline data (Table 3). In the Hyal group, 50% (4/8) of the dogs were classified as severe degree of OA (grades III to IV) and 50% as moderate degree (grade II) of OA. In the Control group, 25% (2/8) of the dogs were classified as severe degree of OA (grades III to IV) and 75% as mild to moderate degree (grades I to II) of OA (Table 3).

**Figure 1:**
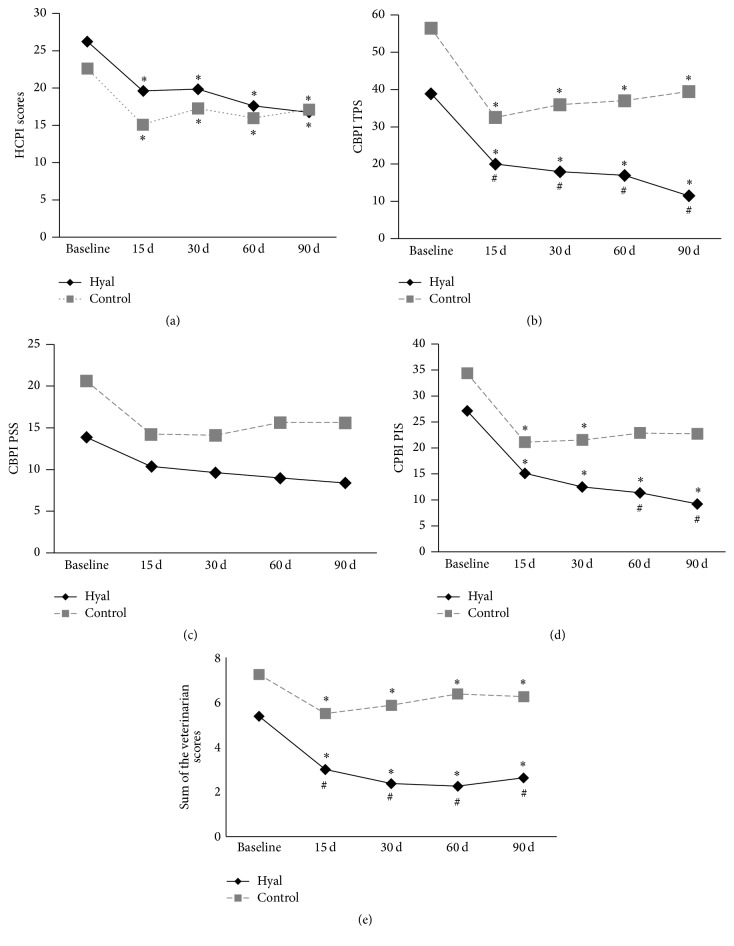
Helsinki Chronic Pain Index (HCPI) scores (a), Canine Brief Pain Inventory (CBPI) scores referring to total pain scores (TPS) (b), pain severity scores (PSS) (c), pain interference scores (PIS) (d), and the scores evaluated by the veterinarian (e) prior to treatment (i.e., baseline) and over time in dogs treated with intra-articular hyaluronic acid (Hyal, *n* = 8) or traditional conservative treatment (Control, *n* = 8). ^*∗*^Significantly different from baseline values. ^#^Significantly different from Control group. ANOVA with posttest Tukey-Kramer multiple comparisons test (*P* < 0.05).

Compared with the baseline, lower scores were observed in both groups over the 90 days in the veterinarian evaluation (*P* < 0.001), the HCPI (*P* < 0.001), the total CBPI (*P* < 0.001), and the PIS-CBPI (*P* < 0.001 Hyal; *P* = 0.019: Control). In the comparison between groups, lower scores were observed from 60 to 90 days in the CBPI scores (total and PIS) in the Hyal group compared to the Control group. The Hyal group exhibited lower scores from 15 to 90 days compared with the Control group in the veterinarian evaluation (Tables 4 and 5 and Figure 1).

Overall, the scores (HCPI, CBPI, and veterinarian assessment) decreased around 30% from 15 to 90 days in the Control group. In the Hyal group, the greatest improvements (≥40%) in the CBPI scores (total, PSS, and PIS), and HCPI scores were noted from 30 to 90 and 60 to 90 days after injection, respectively.

Analgesic intervention was not required during the evaluation period.

Correlations with body weight, age, and clinical severity at trial entry were not significant. In the Hyal group, age was correlated with mean baseline HCPI score (SR = 0.90; *P* = 0.002) and HCPI score at the end of the trial (SR = 0.73; *P* = 0.03).

Clinical improvement was observed in 50% (4/8) of the animals in the Control group and 100% (8/8) of the animals in the Hyal group, through the total-CBPI evaluation, and the difference was considered significant (*P* = 0.028). In the evaluation using the HCPI, the incidence of clinical improvement was observed in 25% (2/8) of the animals in the Control group and 50% (4/8) of the animals of the Hyal group for the evaluation with CBPI, with no difference between groups (*P* = 0.396).

The total indices evaluated by the owners using total CBPI showed an average of 56.4% and 29.6% clinical improvement in animals from the Hyal and Control groups, respectively, a statistical difference being detected between groups (*P* = 0.010). Using the HCPI, improvement indices were 34% and 24% in animals from the Hyal and Control groups, respectively, with no statistical difference between treatments (*P* = 0.505).

With respect to the overall impression of the dogs at the end of treatment, 75% of owners evaluated the quality of life as very good to excellent in the dogs of the Hyal group, while in the Control group the majority of owners (75%) evaluated the quality of life as good; a statistical difference was detected between the groups (*P* = 0.046).

Regarding adverse effects, three animals (two in the Hyal group and one in the Control group) demonstrated signs of pain in the first 24 h after the IA injection. Alterations in the digestive system were not observed, and the gastric mucosa protector was not required.

## 4. Discussion

This is the first study to compare the use of IA injection of HA with TCT in dogs with OA in the hip joint. Our results suggest that both treatments reduced the clinical signs of hip OA; however greater improvement was achieved in dogs with HA given by intra-articular injection, confirming the hypothesis of the study.

The HA dose administered was based on previous studies in dogs [20, 28]. There is no consensus in the current literature with respect to the required number of IA applications of HA to obtain satisfactory results. Studies developed with humans have reported success with a single IA application [29, 30] and with multiple applications at weekly intervals [15, 31]. In dogs undergoing surgical correction of patellar luxation, a single IA application of HA at the end of surgery resulted in similar effects to the application of two doses at weekly intervals [20]. In humans, similar results were reported by Kolarz et al. [32], who demonstrated that, in treatment with HA intra-articular injection, single or multiple applications resulted in similar beneficial effects in patients with signs of OA. In the current study, we opted for the single application of HA without the use of other associated medications due to the lack of published data on this treatment for dogs with HD/OA. However, the efficacy of single and multiple injections of HA in dogs with OA would be an interesting subject for further studies.

Clinical reports have demonstrated beneficial effects on pain, function, and patient global assessment especially 5–13 weeks following IA HA injection [31, 33]. The majority of studies have shown a percent improvement from baseline of 28–54% for pain and 9–32% for function [13, 15, 17]. Similarly, in the current study, the most significant decreases in the owner-based assessment scores (≤40%) were observed from 30 to 90 days (4 to 12 weeks) after HA injection. Clinical improvements in CBPI and in HCPI scores were seen in both treatment groups. However, significant differences in both CBPI (total and PIS scores) and HCPI scores were only seen from 60 to 90 days in the Hyal group in comparison to the Control group. These findings are in accordance with the results of other investigations that described a long-lasting benefit of HA [30, 31, 33]. In addition, it is possible that the treatment with carprofen given to the Control group could mask the differences between groups in the 30-day period following IA injection. The treatment prescribed for the Control group was based on the treatment commonly used by veterinarians to reduce clinical signs of HD in dogs. The anti-inflammatory chosen was carprofen, as it is one of the most widely studied NSAIDs in dogs with OA [34, 35], which several clinical studies have confirmed regarding the safety of this drug for periods exceeding 15 days of treatment [11, 34].

In the present study, the total indices evaluated by the owners of the dogs using the CBPI demonstrated a greater clinical improvement in animals treated with HA (56.4%) compared to the Control group (29.6%). Similar results have been reported in humans with knee OA, with reports of higher efficiency in the reduction of joint pain over 12 weeks in patients treated with HA compared to those treated with triamcinolone, both via IA [30].

It is known that factors such as body weight, age, and degree of cartilage degeneration can influence the efficacy of OA treatment [31]. In the current study, the randomization of dogs between treatments resulted in groups that were not completely homogeneous. Thus, there was a difference with regard to body weight, age, and degree of OA. In the Control group the sample consisted of a superior proportion of larger (<20 kg) and younger (≤7 years) dogs than the Hyal group. Body weight can influence degenerative joint disease by affecting the stresses on joints and smaller dogs may be better able to compensate for orthopedic disease compared to larger dogs [26]. Using TCT, only two of six larger dogs exhibited an overall clinical improvement with treatment. Interestingly, all the larger dogs treated with HA achieved an improvement of 30% or more at 12 weeks. This finding may be attributed to the viscoelastic properties of HA, which act as a lubricant and shock absorber [13]. These effects are related to a reduction in sensitivity to mechanical forces of stretch-activated channels present in the membrane of joint mechanonociceptors [20].

As expected, the older dogs exhibited a superior degree of OA (grades III and IV) compared to younger dogs. In general, the older dogs showed a clinical improvement with both treatments. However, a greater improvement was seen in the dogs treated with HA. This result can be explained by the various mechanisms of HA, including restoration of the elastic and viscous properties of the synovial fluid and anti-inflammatory, antinociceptive, and chondroprotective effects. Experimental animal models of OA have reported that intra-articular HA injections may decrease degradation of the cartilage matrix. Zhang et al. [36] showed that HA injections provided better cartilage and synovial conditions than a placebo in rats submitted to surgically-induced OA. Another study demonstrated that cross-linked HA alone or in combination with ropivacaine or triamcinolone produced a significant improvement in knee articular cartilage in a rabbit model of collagenase-induced knee osteoarthritis [37].

Another important aspect is the correlation between clinical improvement indices and the quality of life for the dogs. In animals treated with HA, 75% of owners considered the quality of life of the animals after treatment to be very good to excellent. These results corroborate previous studies reported in humans, which attributed the best quality of life indices to patients who responded favorably to analgesic therapies, demonstrating a direct correlation between the relief of chronic pain and improved quality of life [15, 30].

One of the most relevant aspects of the present study refers to the advantages conferred by treatment with HA compared to TCT in dogs with HD/OA. From a clinical point of view, the most interesting factor concerns the possibility of using HA in isolation, without the need for additional analgesia 90 days after the IA administration, since NSAIDs may induce adverse effects when administered for prolonged periods, in addition to representing a risk factor for animals with a history of clotting disorders or renal and digestive diseases [11]. In parallel, the administration of a single IA injection of HA is a highly practical form of treatment, eliminating the daily oral administration of a nutraceutical or NSAID, a limiting factor in the treatment of dogs reluctant to swallow medication or that demonstrate aggressive behaviour. However, despite the favorable results obtained in this study, the therapeutic choice should be made on individual basis, carefully weighing the relative benefits and disadvantages of the treatment.

Although rare, complications of IA injections, such as joint infection, pain following injection, and skin pigment may occur. In the present study, no serious adverse events were noted. Of the 16 dogs evaluated, only three owners reported signs compatible with discomfort in the first 24 hours following the IA injection and this was of a temporary nature and did not represent clinical relevance. This result supports previous studies that reported minimal adverse effects following IA injection in humans [15, 31] and dogs [20, 28]. Another unfavorable aspect is related to the requirement of general anaesthesia for the hip infiltration, which may represent a limitation factor for some dogs.

Pain evaluation in animals is a difficult challenge, due to the impossibility of verbal expression, which makes the measurement of this parameter extremely subjective on the part of the evaluators. Many studies have been developed in order to improve the scales used in the evaluation of chronic pain, involving not only the evaluation by the researcher [21] but also the use of multifactorial questionnaires directed to the owners of the animals, taking into account behavioral change, walking ability, signs of pain, and quality of life [25–27]. In the present study, the experimental design was double-blind to minimize any bias in the results on the part of the researcher or the owners of the animals. In addition, the clinical evaluation of the dogs was performed by a veterinary orthopedic surgeon experienced in the evaluation of animals with OA, who has previously participated in other studies involving dogs with OA. In addition, as the pain scales are considered extremely subjective, in the present study, all the measurements were performed by the same trained observer, in an attempt to control the variability between different assessors.

The questionnaires used in the present study have been validated for evaluation of chronic pain in dogs with OA [25–27]. The results reported by the owners demonstrated greater differences between groups for evaluations using the CBPI. In dogs with OA, this questionnaire was capable of identifying clinical improvement in animals treated with NSAIDs compared to a placebo, suggesting it to be a viable method for evaluating the clinical evolution of dogs with OA [26, 27]. Differences were not found between the groups over time when using the HCPI. Similar results were observed by Teixeira [38] who used this questionnaire for the evaluation of clinical signs in dogs with HD subjected to different treatments. In this study, many of the owners demonstrated some degree of difficulty in completing HCPI questionnaire, which may have affected the outcome of the evaluation. Furthermore, although the owner-based questionnaires used in this study have been validated, it is possible that more reliable results could be obtained with an objective outcome measure such as kinetic force plates.

This study has some limitations. Among them, we can highlight the small number of animals evaluated. With a larger sample more reliable results could be demonstrated. Furthermore, the number of animals involved in the study was restricted due to the inclusion criteria and also the need for the owner's consent. Some of the owners refused to participate in the study due to the necessity of general anaesthesia in order to access the hip joint; many of the dogs were of an advanced age which increased the concern of the owners. Another limiting factor of the study is the large heterogeneity of the studied population. Although significant differences were not found in the demographic data, there was considerable individual variation with regard to body weight (07 to 35 kg), age (1.5 to 15 years), and breeds evaluated. Initially it was expected that body weight would be similar due to the higher incidence of the HD in large breeds. However, contrary to expectations, the breeds were diverse. Furthermore, despite the selection of animals with chronic pain associated with hip OA, the intensity of clinical signs and duration of disease progression differed between individuals. However, these differences are inherent in clinical studies, and form part of those variables that cannot be fully controlled and reveal the reality of clinical practice. In addition, our study design did not include a third group for evaluation of both treatments in combination. While HA and oral nutraceuticals have different mechanisms of action, simultaneous administration of the two drugs could result in a synergistic effect. Besides this, it is possible that saline in isolation might have had a positive effect, as reported by Gaustad et al. [39]. Thus, the inclusion of a fourth group using only TCT could lead to a better understanding of how the different treatments act in dogs with hip OA.

It was concluded that both treatments reduced the clinical signs of hip OA; however, the best results were obtained with IA injection of HA, which may represent a viable alternative for dogs with OA induced by HD. Therefore, additional larger and long-term (up to one year) studies are needed to support these results. Additionally, future research should focus on cost effectiveness of therapy and relation between molecular weight and effectiveness.

## Figures and Tables

**Table 1:** Clinical scoring system for assessing dogs.

Criterion	Grade	Clinical evaluation
Pain on palpitation	0	No signs of pain on palpation of the affected joint
	1	Slight signs of pain on palpation of the affected joint, the dog turns its head in recognition
	2	Moderate signs of pain on palpation of the affected joint, the dog pulls the limb as a defense reaction
	3	Severe signs pain on palpation, the dog vocalizes or becomes aggressive
	4	The dog does not allow palpation

Lameness	0	Normal, no lameness
	1	Mild lameness, not very difficult to move
	2	Clear lameness, not moving freely
	3	Obvious lameness when walking
	4	Severe lameness preventing the dog from supporting weight on the affected limb

Ability to jump	0	Jumps normally
	1	Jumps with care
	2	Jumps with some difficulty
	3	Jumps or rises with great difficulty
	4	Does not try because of the difficulty/pain

Ability to climb stairs	0	Goes up and down the stairs normally
	1	Slightly careful, uses both paws successively
	2	Sometimes uses both feet at the same time, evidently does not move freely
	3	Goes up the stairs like a rabbit at all times, goes up the stairs with great difficulty
	4	Does not try to climb because of the difficulty/pain

**Table 2:** Mixed model for repeated measurements of Helsinki Chronic Pain Index (HCPI) scores, Canine Brief Pain Inventory (CBPI, total) scores, and the scores evaluated by the veterinarian prior to treatment (i.e., baseline) and over time in dogs treated with intra-articular hyaluronic acid (Hyal, *n* = 8) or traditional conservative treatment (Control, *n* = 8).

Effect	*F* test	*P*
*HCPI*		
Time	20.88^*∗*^	<0.001
Interaction time × treatment	1.80	0.18

*CBPI*		
Time	20.44^*∗*^	<0.001
Interaction time × treatment	1.28	0.30

*MedVet scores*		
Time	22.68^*∗*^	<0.001
Interaction time × treatment	4.62	0.003

^*∗*^Significant differences over time (*P* < 0.05).

**Table 3:** Baseline characteristics of the study population.

Patient data	Hyal (*n* = 8)	Control (*n* = 8)	*P* (value)
Body weight (kg)^a^	18 ± 8	22.7 ± 10	0.14

Age (years)^a^	8 ± 5	4.6 ± 2.3	0.09

Male/female	4/4	3/5	

Clinical severity			
Grade D	7/8	7/8	
Grade E	1/8	1/8	

Affected hip joint			
Bilateral	7/8	7/8	
Unilateral	1/8	1/8	

Breeds			
Labrador	1/8	3/8	
Crossbreed	2/8	2/8	
Boxer	1/8	2/8	
Border Collie	2/8	1/8	
Lhasa Apso	2/8		

Estimated duration of symptoms (years)^a^	1.6 ± 0.5	1.8 ± 0.8	0.13

HCPI at baseline^a^	26.2 ± 8	22.6 ± 6	0.35

CBPI at baseline^a^	41 ± 16	55 ± 16	0.19

Veterinary index at baseline^a^	5.3 ± 1.5	7.25 ± 2	0.11

Degree of OA at baseline^a^	2.6 ± 0.7	2.0 ± 0.7	0.19

^a^Values expressed as mean ± SD.

**Table 4:** Patient characteristics of sixteen dogs treated with intra-articular hyaluronic acid (Hyal, *n* = 8) or traditional conservative treatment (Control, *n* = 8).

Group/number of dog	Degree of OA	Weight (kg)	Age (years)	Breed	Improvement index CBPI (%)	Improvement index HCPI (%)
Hyal						
01	4	27	08	Labrador	34	27
02	2	19	02	Crossbreed	41	25
03	3	25	09	Boxer	69	31
04	2	22	1.5	Border Collie	79	98
05	2	12	15	Crossbreed	60	47
06	3	07	11	Lhasa Apso	49	0.5
07	2	09	11	Lhasa Apso	39	17
08	3	24	07	Border Collie	50	28

Control						
01	1	10	02	Crossbreed	51	28
02	2	30	05	Labrador	28	26
03	2	15	05	Crossbreed	10	20
04	3	28	07	Boxer	38	30
05	1	22	02	Border Collie	34	22
06	3	25	08	Labrador	23	32
07	2	24	02	Boxer	44	26
08	2	28	06	Labrador	19	27

**Table 5:** Helsinki Chronic Pain Index (HCPI) scores, Canine Brief Pain Inventory (CBPI) scores referring to total pain scores (TPS), pain severity scores (PSS), pain interference scores (PIS), and the scores evaluated by the veterinarian prior to treatment (i.e., baseline) and over time in dogs treated with intra-articular hyaluronic acid (Hyal, *n* = 8) or traditional conservative treatment (Control, *n* = 8).

Variables	Groups	Time of assessment					^*∗*^ *P* value
		Baseline	15 days	30 days	60 days	90 days	
HCPI score	Hyal	26 ± 8	19 ± 10^*∗*^	19 ± 11^*∗*^	17 ± 11^*∗*^	16 ± 11^*∗*^	*P* < 0.001
% change			28%	27%	40%	43%	
HCPI score	Control	22 ± 6	15 ± 3^*∗*^	17 ± 3^*∗*^	16 ± 4^*∗*^	17 ± 5^*∗*^	*P* < 0.001
% change			31%	22%	29%	24%	

CBPI TPS	Hyal	41 ± 16	25 ± 17^*∗*^	22 ± 18^*∗*^	20 ± 18^*∗*#^	17 ± 16^*∗*#^	*P* < 0.001
% change			36%	50%	54%	64%	
CBPI TPS	Control	55 ± 16	35 ± 12^*∗*^	35 ± 13^*∗*^	38 ± 11^*∗*^	38 ± 11^*∗*^	*P* < 0.001
% change			32%	31%	28%	28%	
^#^ *P* value					0.03	0.02	

CBPI PSS	Hyal	13 ± 5	10 ± 7	9 ± 8	9 ± 7	8 ± 7	
% change			30%	40%	40%	49%	
CBPI PSS	Control	18 ± 6	14 ± 5	14 ± 4	15 ± 5	15 ± 5	
% change			31%	33%	30%	27%	

CPBI PIS	Hyal	27 ± 12	15 ± 12^*∗*^	12 ± 10^*∗*^	11 ± 9^*∗*#^	9 ± 9^*∗*#^	*P* < 0.001
% change			30%	40%	40%	49%	
CPBI PIS	Control	34 ± 11	21 ± 8^*∗*^	21 ± 6^*∗*^	22 ± 7	22 ± 5	*P* = 0.019
% change			30%	30%	31%	32%	
^#^ *P* value					0.02	0.02	

Vet score	Hyal	5 ± 1	3 ± 2^*∗*#^	2 ± 1^*∗*#^	2 ± 1^*∗*#^	2 ± 1^*∗*#^	*P* < 0.001
% change			42%	56%	64%	57%	
Vet score	Control	7 ± 2	5 ± 1^*∗*^	6 ± 2^*∗*^	6 ± 2^*∗*^	6 ± 1^*∗*^	*P* < 0.001
% change			24%	20%	13%	13%	
^#^ *P* value			0.01	0.001	0.001	0.002	

Note: data are expressed as mean ± standard deviation (SD). ^*∗*^Significantly different from baseline values. ^#^Significantly different from Control group. ANOVA with posttest Tukey-Kramer multiple comparisons test (*P* < 0.05).
